# Associations between healthcare utilization and access and diabetic retinopathy complications using *All of Us* nationwide survey data

**DOI:** 10.1371/journal.pone.0269231

**Published:** 2022-06-15

**Authors:** Alison X. Chan, John J. McDermott IV, Terrence C. Lee, Gordon Y. Ye, Bita Shahrvini, Bharanidharan Radha Saseendrakumar, Sally L. Baxter

**Affiliations:** 1 Viterbi Family Department of Ophthalmology and Shiley Eye Institute, University of California San Diego, La Jolla, CA, United States of America; 2 UCSD Health Department of Biomedical Informatics, University of California San Diego, La Jolla, CA, United States of America; Oxford University Hospitals NHS Foundation Trust, UNITED KINGDOM

## Abstract

**Purpose:**

Inadequacies in healthcare access and utilization substantially impact outcomes for diabetic patients. The *All of Us* database offers extensive survey data pertaining to social determinants that is not routinely available in electronic health records. This study assesses whether social determinants were associated with an increased risk of developing proliferative diabetic retinopathy or related complications (e.g. related diagnoses or procedures).

**Methods:**

We identified 729 adult participants in the National Institutes of Health *All of Us* Research Program data repository with diabetic retinopathy (DR) who answered survey questions pertaining to healthcare access and utilization. Electronic health record data regarding co-morbidities, laboratory values, and procedures were extracted. Multivariable logistic regression with bi-directional stepwise variable selection was performed from a wide range of predictors. Statistical significance was defined as p<0.05.

**Results:**

The mean (standard deviation) age of our cohort was 64.9 (11.4) years. 15.2% identified as Hispanic or Latino, 20.4% identified as Black, 60.6% identified as White, and 19.3% identified as Other. 10–20% of patients endorsed several reasons for avoiding or delaying care, including financial concerns and lack of access to transportation. Additional significant social determinants included race and religion discordance between healthcare provider and patient (odds ratio [OR] 1.20, 95% confidence interval [CI] 1.02–1.41, p = 0.03) and caregiver responsibilities toward others (OR 3.14, 95% CI 1.01–9.50, p = 0.04).

**Conclusions:**

Nationwide data demonstrate substantial barriers to healthcare access among DR patients. In addition to financial and social determinants, race and religion discordance between providers and patients may increase the likelihood of PDR and related complications.

## Introduction

Diabetic retinopathy (DR) is a leading cause of blindness among working-age adults in the United States and is the most common microvascular complication of diabetes [1]. Despite American Diabetes Association recommendations for a minimum of one dilated eye exam per year for patients with diabetes, only 35% to 60% of patients in the United States have been reported to adhere to these recommendations [2, 3]. Survey data from the National Health Interview Survey indicates that nearly one-half of adults aged 40–64 diagnosed with diabetes have not had contact with an eye doctor in the past 12 months [4]. Reasons for poor utilization of diabetic healthcare services include socioeconomic barriers to care, including accessibility to eye care providers and insurance coverage [5]. Poor patient education may also play a role; a cross-sectional study of adults participating in the National Health and Nutritional Examination Surveys found that several patients with retinopathy had limited contact with diabetic education specialists in the past year [6]. These barriers to diabetic eye care have significant implications for vision-related morbidity and may lead to advancement to proliferative diabetic retinopathy and related complications including neovascular glaucoma, retinal detachment, and vitreous hemorrhage.

The National Institutes of Health (NIH) *All of Us* Research Program (“*All of Us”*) launched in May 2018 and represents a nationwide initiative to create a database reflecting the increasing diversity of the United States. Participants are surveyed regarding race, ethnic group, age, sex, access to care, income, and educational attainment. Underrepresented populations are prioritized for physical measurements and biospecimen collections [7]. To date, the *All of Us* database offers electronic health record and survey data for more than 364,000 participants, more than 80% of which are underrepresented minorities in biomedical research [8]. By approximately 2024, the program is expected to enroll nearly 1 million participants [7].

Although prior studies have investigated the socioeconomic risk factors implicated in DR progression, less is known about the patterns of healthcare utilization among DR patients, particularly those from backgrounds traditionally underrepresented in clinical research studies. In this study, we leverage the size and diversity of the *All of Us* research database to identify social determinants associated with increased risk of developing PDR or related complications.

## Methods

### Study population

The goals, recruitment methods, and scientific rationale for *All of Us* have been described previously. *All of Us* includes surveys, electronic health record (EHR) domains, and physical measurements (PM) that can be accessed and analyzed using the *All of Us* Researcher Workbench, a cloud-based platform. Survey details can be found in the Survey Explorer in the Research Hub, a website designed to support researchers [9]. Each of the surveys includes branching logic. All surveys other than an initial basic demographics survey are optional and may be skipped by the participant. PM recorded at enrollment include blood pressure, height, weight, heart rate, waist and hip measurement, wheelchair use, and current pregnancy status. EHR data regarding medical conditions, procedures, and labs and measurements were linked for consented participants. Data collection was approved by the *All of Us* Institutional Review Board. All three data types (survey, PM, and EHR) are mapped to the Observational Health and Medicines Outcomes Partnership (OMOP) common data model v5.2 maintained by the Observational Health and Data Sciences Initiative (OHDSI) collaborative [https://www.ohdsi.org/].

*All of Us* performed data transformations across each participant record to protect participant privacy [10]. These transformations include: data suppression of codes with a high risk of identification; generalization of categories such as age, sex at birth, gender identity, sexual orientation, and race; and date shifting by a random (less than one year) number of days. The *All of Us* Registered Tier Curated Data Repository (CDR) Data Dictionary contains formal documentation on privacy implementation and creation of the CDR [11]. The Researcher Workbench provides access to Registered Tier data and enables researchers to select groups of participants (Cohort Builder), save health information about cohorts (Dataset Builder), and analyze data using Jupyter Notebooks (Notebooks). Within the Notebook environment, high-powered queries and analyses can be performed using R and Python 3 programming languages. Secondary analyses of de-identified data included in *All of Us*, such as that presented here, is considered non-human subjects research.

At the time of analysis, there were over 364,000 adult participants in *All of Us*. Our study cohort consisted of adult (age 18 years and above) participants with diabetic retinopathy due to Type 2 diabetes who answered the survey on the All of Us platform titled “Healthcare Access and Utilization. This is a 42 question survey that asks various questions about a participant’s access to and utilization of healthcare; a complete list of survey items and answer choices as they appear on *All of Us* is available in the Appendix in S1 File (“*All of Us* Research Program Survey on Healthcare Access and Utilization” in S1 File). The diagnosis of diabetic retinopathy was determined through International Classification of Diseases (ICD) codes related to the SNOMED code for diabetic retinopathy due to Type 2 diabetes (S1 Table in S1 File). Since *All of Us* does not currently include children in its database, and Type 1 diabetes most commonly presents in childhood, patients with Type 1 diabetes were excluded.

### Data processing

The Researcher Workbench was used to extract relevant data for the analysis. First, the cohort was defined as described above. Concept sets are a standard term in the OMOP common data model to indicate which codes and values comprise specific variables used in analysis. Concept sets for the outcome and each predictor were built in the Workbench by selecting relevant procedure and diagnosis codes used by medical professionals (e.g., ICD and/or SNOMED codes for conditions, Logical Observation Identifiers Names and Codes [LOINC] for measurements and observations, and Current Procedure Terminology [CPT] codes for procedures). The outcome of interest was proliferative diabetic retinopathy (PDR) and related diagnoses and procedures. The outcome was defined by searching for the following codes within the *All of Us* Researcher workbench: diagnosis codes which included “proliferative diabetic retinopathy,” diagnoses related to PDR (e.g., diabetic tractional retinal detachment, neovascular glaucoma, and diabetic vitreous hemorrhage), and procedure codes for PDR-related complications (e.g., photocoagulation, vitrectomy, membrane peeling, and repair of diabetic traction retinal detachment). A full list of procedure and diagnosis codes that were used to define the outcome of proliferative diabetic retinopathy and related complications is available in S5 Table in S1 File.

Concept sets were created in the *All of Us* Researcher Workbench for the independent variables in our analysis. These variables include demographics, micro- and macrovascular conditions associated with diabetic retinopathy (e.g., diabetic nephropathy and peripheral neuropathy), lab values related to diabetes severity (e.g., glomerular filtration rate, glycosylated hemoglobin A1c, and creatinine), and healthcare access and utilization survey data regarding social determinants. A full list of independent variables used in this study is available in S6 Table in S1 File. These concept sets were used to create “datasets,” or tables, containing data about a cohort that can be exported for analysis. To establish a temporal relationship between predictor and outcome, patients were included only if the predictors preceded the outcome diagnosis of PDR or related complications. Subsequent analyses were performed in an R notebook within the *All of Us* Workbench environment. All data extraction and cleaning procedures can be found in the referenced R notebook in our publicly available workspace [12].

### Data analysis and modeling

Descriptive statistics of the *All of Us* DR study cohort were generated regarding age, gender, and race (Table 1).

**Table 1:** Demographic data of DR participants with healthcare access and utilization survey data. Demographics of the general *All of Us* adult population and the general United States (US) population based on the 2020 census are included for reference regarding representativeness of the cohort.

	DR cohort that answered healthcare access and utilization survey (N = 729)	*All of Us* adult population (N = 302,601)	US population in the year 2020 (N = 329,484,119)
**Age** (Mean, SD)	64.9 (11.41)	53.34 (16.73)	N/A
**Gender** (n, %)			
Male	341 (46.78%)	116,146 (38.38%)	162,211,577 (49.23%)
Female	388 (53.22%)	186,455 (61.62%)	167,272,542 (50.77%)
**Self-Reported Race** (n, %)			
Black or African American	149 (20.44%)	66,434 (21.95%)	39,839,863 (12.09%)
White	442 (60.63%)	163,655 (54.08%)	206,619,960 (62.71%)
Other	138 (18.9%)	72,512 (23.97%)	83,024,296 (25.20%)
**Self-Reported Ethnicity** (n, %)			
Not Hispanic or Latino	603 (82.72%)	241,420 (79.78%)	268,350,627 (81.45%)
Hispanic or Latino	111 (15.23%)	57,961 (19.15%)	61,133,492 (18.55%)

* Original self-reported race categories included Asian, but this category was collapsed together to avoid secondary calculation of cells <20.

** Counts less than 20 (and corresponding percentages) cannot be displayed due to NIH *All of Us* Research Program Data and Statistics Dissemination Policy. In some cases, additional data may be obscured to prevent secondary calculation of these values.

### Analysis of healthcare access and utilization survey responses

**Fig 1:**
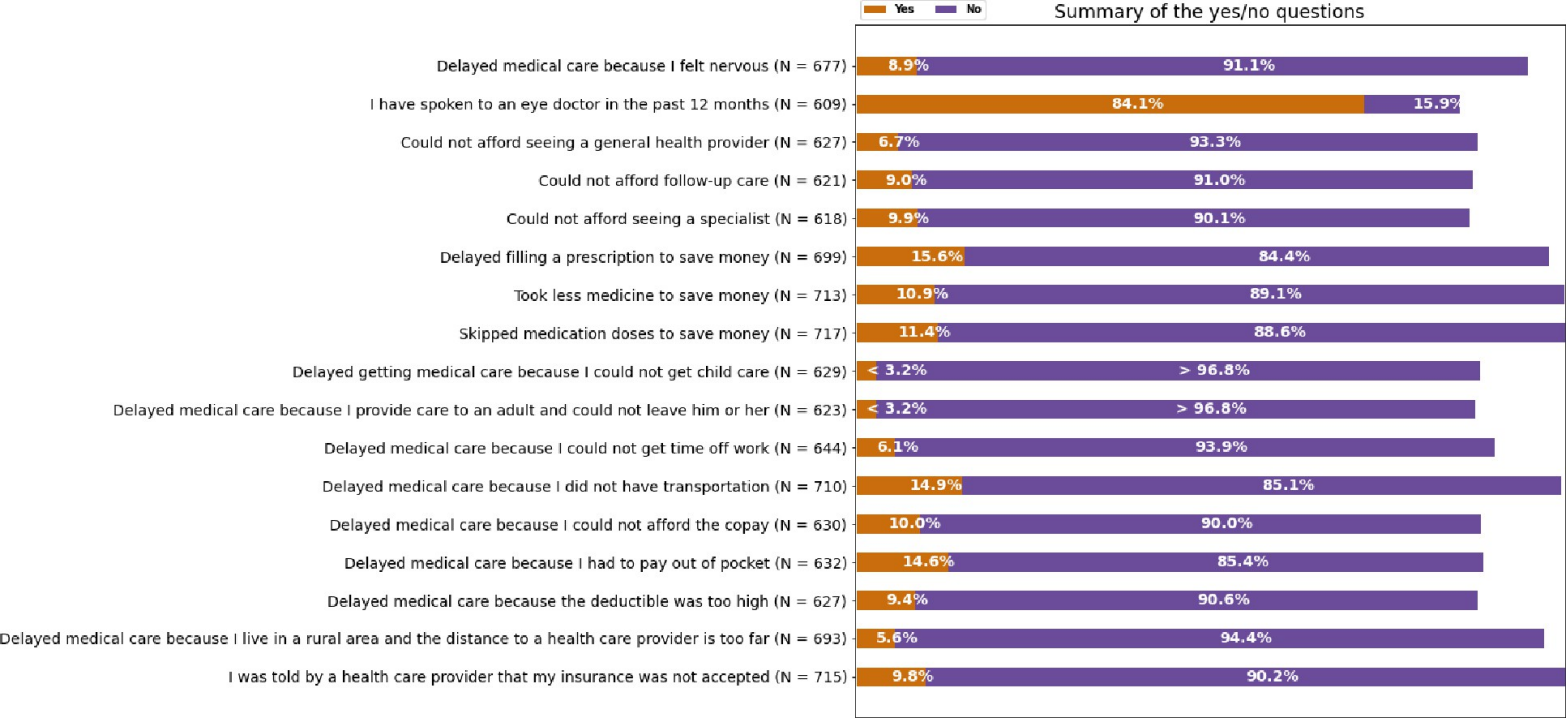
Distribution of responses to questions regarding healthcare access and utilization among patients with diabetic retinopathy in the NIH *All of Us* research program.

Tables were created for patient responses to closed-ended survey questions (e.g., “yes” or “no”), while responses to questions with answer choices were displayed as continuous variables in histogram [13]. A helper function was used to represent frequency distributions of survey responses to the closed-ended questions as a summary bar plot (Fig 1). Counts less than 20 (and corresponding frequencies) are unable to be displayed individually due to *All of Us* data sharing policies. Information on when the survey was collected in relation to the DR or DR complication diagnosis was unable to be obtained since some *All of Us* survey items have historical components that do not delineate specific time periods.

Each survey item had a response rate of 96% or higher. Individual response rates are available in the workspace (12). Regarding imputation methods, for patients who skipped survey questions with categorical answers, we replaced missing values with the mode (i.e., the most common survey response).

### Logistic regression modeling

Logistic regression modeling (bivariate and multivariable) was performed via R using predictors for 729 participants who had all predictor data available [13]. The following R packages were used: *stats*, g*gplot2*, *tibble*, *tidyr*, *readr*, *purrr*, *dplyr*, *stringr*, *forcats*.

Bivariate analyses were performed to determine statistically significant variables. Bivariate (crude) odds ratios (OR) and 95% confidence intervals (CIs) were calculated for all predictors.

Predictors included demographic information (gender, race, ethnicity, etc.), all variables from healthcare utilization and access surveys (“*All of Us* Research Program Survey on Healthcare Access and Utilization” in S1 File), and lab values (glycosylated hemoglobin A1c and creatinine). Data on predictors were only included if they were present before the outcome (i.e. diagnosis of PDR or related complication). For imputation, replacement of categorical data with the mode was used, whereas missing continuous data were replaced with the mean. Analysis of missing data revealed that the values were missing completely at random. More information regarding the number and percentage of missing values for each variable can be found in S4 Table in S1 File.

Multivariable logistic regression modeling was performed to determine which predictors were significantly associated with increased odds of developing PDR or related complications. We evaluated correlation coefficients among predictor variables with the objective of removing highly correlated variables (correlation coefficient >0.9). Correlation analyses with both the Kendall rank and Spearman’s rank method revealed that none of our predictor variables were highly correlated. We used bi-directional stepwise variable selection using the Akaike information criterion (AIC). Using the best-performing multivariable model, we calculated and reported adjusted odds ratios, their 95% CIs, and associated p-values. Statistical significance was defined as p<0.05.

## Results

### Healthcare access and utilization among adults with diabetic retinopathy

We identified a cohort of 729 adults (Table 1). The majority (n = 388, 53.2%) were female. The mean (standard deviation, SD) age of participants was 64.9 (11.4) years. Black participants (n = 149) represented 20.4% of the cohort while Hispanic or Latino participants (n = 111) represented 15.2% (Table 1).

229 (31.4%) participants were diagnosed with proliferative diabetic retinopathy or related complications (Table 2). Common ophthalmic complications included vitreous hemorrhage (n = 56, 7.7%) and requirement for photocoagulation (n = 35, 4.8%). 141 (19.3%) had concurrent kidney disorder from diabetes, and less than 20 (<8.7%) had peripheral neuropathy associated with diabetes.

**Table 2:** Distribution of patients with complications of diabetic retinopathy (n = 229).

Complications of diabetic retinopathy	Number of patients
Proliferative diabetic retinopathy due to type 2 diabetes mellitus	164 (7.16%)*
Vitreous hemorrhage	56 (24.5%)
Tractional retinal detachment with type 2 diabetes mellitus	<20^†^
Treatment of extensive or progressive diabetic retinopathy, including photocoagulation	35 (15.3%)
Repair of complex retinal detachment or diabetic traction retinal detachment	<20
Pars plana vitrectomy with endolaser panretinal photocoagulation	<20

* Percentages may not add to 100 due to some patients having multiple complications.

† Counts less than 20 are not shared in accordance with *All of Us* data reporting policies.

Regarding utilization of ophthalmic services, 97 (13.3%) survey respondents endorsed that they had not spoken to an eye doctor within the past 12 months. Less than 30% of patients with diabetic retinopathy (208, 28.5%) had one visit to the eye doctor in the past 12 months. In contrast, the majority (n = 449, 61.6%) of participants claimed that they had spoken to any medical specialist in the past 12 months.

Among the factors assessed by the healthcare utilization and access surveys, the inability to afford care was commonly cited as a reason for delaying care. 97 (16.1%) participants reported delaying filling prescriptions to save money, while 82 (11.2%) stated that they skipped their medications altogether to save money. 92 (12.6%) delayed seeking medical care due to having to pay out of pocket (Fig 1).

Besides financial concerns, approximately 9–14% of participants with diabetic retinopathy who answered the survey indicated they faced additional barriers for timely care, such as lack of transportation, inadequate healthcare coverage, or feeling nervous (Fig 1).

### Factors associated with increased risk of proliferative diabetic retinopathy and related complications

Factors were individually analyzed to evaluate potential associations with increased odds of developing PDR and related complications. These factors included demographic characteristics (e.g. race, age, gender, employment status, household income, etc.), co-morbidities (e.g. peripheral neuropathy, proteinuric nephropathy, macro/microalbuminuric nephropathy due to diabetes mellitus, etc.), values for glycosylated hemoglobin A1c, fasting glucose, and creatinine, and healthcare access and utilization data (S5 Table in S1 File). Several factors were significantly associated with increased odds of developing PDR: age, the number of eye doctor visits, kidney disorder due to diabetes, and financial constraints leading to delays in care (Table 3).

**Table 3:** Bivariate crude odds ratios for variables associated with increased odds of developing proliferative diabetic retinopathy (PDR) or related complications.

Variable	N(%) or mean (SD) without PDR (N = 582)	N (%) or mean(SD) with PDR or related complications (N = 147)	Odds ratio (95% Confidence Interval)	P-value
Diagnosed with kidney disorder due to diabetes (those with diagnosis vs. those without diagnosis of kidney disorder due to diabetes)	96 (16.49%)	42 (28.57%)	2.03 (1.32–3.07)	<0.01
Number of eye doctor visits in the past 12 months	0.64 (1.18)	1.28 (1.7)	1.35 (1.20–1.52)	<0.01
Skipped medication to save money**	55 (9.45%)	27 (18.37%)	2.16 (1.29–3.53)	0.0027
Delayed care due to lack of transportation**	74 (12.71%)	32 (21.77%)	1.91 (1.19–3.01)	0.006
Took less medication to save money**	54 (9.28%)	24 (16.33%)	1.91 (1.12–3.17)	0.015
Delayed care due to caregiver responsibilities**	<20*	<20*	3.18 (1.12–8.69)	0.02
Age (years)	65.35 (11.17)	63.11 (12.21)	0.98 (0.97–0.99)	0.034
Delayed care due to having to pay out of pocket**	66 (11.34%)	26 (17.69%)	1.68 (1.01–2.73)	0.04
Delayed care due to living in a rural area where distance to the health care provider is too far**	26 (4.47%)	<20*	2.07 (1.01–4.07)	0.04
Mean creatinine value	1.21 (1.23)	1.48 (1.64)	1.13 (1.00–1.28)	0.04
Delayed care due to differences in race, religion, or language between patient and provider**	423 (72.68%)	122 (82.99%)	1.18 (1.01–1.37)	0.04

* In accordance with *All of Us* data sharing policies, counts less than 20 are unable to be reported; counts and percentages may not add up as expected due to counts <20 being assigned the value 20 in order to decrease the risk of identification in this cohort.

**These categorical variables were derived from *All of Us* surveys, so corresponding values in the cells indicate the number and percentage of individuals indicating a “yes” response to these survey items. The crude odds ratios describe the odds of developing PDR or related complications associated with “yes” responses to each variable.

A multivariable logistic regression model identified variables significantly associated with increased odds of developing PDR (Table 4).

**Table 4:** Multivariable logistic regression model predicting development of proliferative diabetic retinopathy or related complications among adult patients with diabetic retinopathy.

Variable	Adjusted Odds Ratio (95% Confidence Interval)	p-value
Number of eye doctor visits in the past 12 months	1.32 (1.16–1.49)	<0.001
Diagnosed with kidney disorder due to diabetes mellitus (those with diagnosis vs. those without diagnosis of kidney disorder due to diabetes)	1.94 (1.24–3.01)	0.003
Delayed care due to differences in race, religion, or language between patient and provider (“yes” responses vs. “no” responses)	1.20 (1.02–1.41)	0.03
Delayed care due to having to provide care for another adult (“yes” responses vs. “no” responses)	3.14 (1.01–9.50)	0.04
Skipped medication in order to save money (“yes” responses vs. “no” responses)	1.69 (0.97–2.89)	0.06
Delayed care due to inadequate healthcare coverage (“yes” responses vs. “no” responses)	1.35 (0.97–1.95)	0.09
Age (in years)	0.99 (0.97–1.00)	0.09

## Discussion

Vision loss from diabetic retinopathy can be largely prevented with yearly ophthalmic screening and prompt treatment [14]. Several barriers to receiving diabetic eye care have been identified, including poor patient education about diabetes and related microvascular complications and significant out of pocket costs [15]. Using a nationwide database with diverse enrollment, this study represents an exploratory analysis of patterns of healthcare utilization among those with diabetic retinopathy. We specifically studied a cohort of patients with diabetic retinopathy to assess patients who are at higher risk of vision morbidity or an increased need for frequent monitoring.

First, we found that adherence to diabetic care guidelines remains low. A prior study of eye exam visits showed that only 23.5% of diabetic patients meet the American Diabetes Association (ADA) recommendations for annual eye exams despite adequate patient education [16]. In our study cohort, with enrollment dates ranging from 2018–2020 and survey response rates exceeding 96%, less than 30% of patients with an established diagnosis of diabetic retinopathy reported having one visit to the eye doctor in the past 12 months preceding the survey. This finding reiterates the large gap in diabetic care and is especially concerning given that our study patients had a known diagnosis of diabetic retinopathy and therefore are at greater risk of vision-threatening disease compared to a general diabetic population.

Second, our analysis of healthcare access and utilization survey data found that financial concerns and the costs of care were relatively common barriers to care among patients with diabetic retinopathy and were independently associated with PDR or related complications. This reaffirms prior studies showing that financial barriers are one of the primary barriers to diabetic care [17]. There is a strong need for new strategies to provide affordable and accessible diabetic care for patients, since high costs of diabetic care correlates with increased medication non-adherence and can lead to poor clinical outcomes [18]. One such strategy is the implementation of telemedicine, a cost-effective alternative to face-to-face ophthalmology visits that overcomes patients’ geographical and financial barriers [19]. Teleophthalmology programs assess for DR by transmitting photos taken by primary care health care providers to a reading center for evaluation and have been shown to be safe and accurate alternatives to traditional diabetic retinopathy screening [20]. By saving time and travel costs while eliminating the need to schedule additional appointments with an ophthalmologist, telemedicine can improve ophthalmic care access in remote areas and utilization among vulnerable populations. Already, a substantial increase in telemedicine utilization has been observed during the COVID-19 pandemic, as payers expand coverage and privacy restrictions have been relaxed [21]. As telemedicine technology continues to improve and adapt to the needs of ophthalmology services, virtual DR screenings may find a more permanent role.

Discordance in race, religion, and language between patient and provider emerged as a statistically significant predictor of PDR and related complications in our multivariable model, a novel finding that to our knowledge has not been previously reported in the context of diabetic retinopathy or ophthalmic care more generally. This survey item asked participants whether they have delayed or avoided care altogether because their providers differed in any of the following ways: race, religion, or native language. Despite efforts to address racial disparities in healthcare, minorities continue to have lower rates of healthcare utilization, and a known contributing factor to disparities in utilization is patient and provider race concordance. Lack of racial concordance between physicians and patients has been linked to poor health outcomes among patients requiring cancer, pain, and diabetes management [22]. Race discordance between patients and physicians is associated with lower patient satisfaction rating and lower rates of adherence to prescribed medications and medical interventions [23]. Other studies have found that minority patients prefer the care they receive from minority physicians, as cultural similarities promote positive physician attitudes and improved communication (including information giving and participatory decision-making) [24]. Promoting minority physician representation should be prioritized in the context of diabetic care, especially since minority populations are known to be disproportionately affected by diabetes. Strategies to address the disparities arising from patient-physician discordance include the continued recruitment and training of underrepresented minorities in medicine. This is especially important for certain medical specialties traditionally known to have higher minority underrepresentation; among the ophthalmology workforce, gender imbalances and minority representation were recently highlighted as a major challenge [25].

Our analysis also demonstrated that the number of eye doctor visits and diabetic kidney disease were associated with increased odds of developing PDR. A higher number of eye visits being associated with PDR likely reflects more frequent monitoring of progressing retinopathy rather than being a cause of developing PDR. This illustrates the well-known limitation of observational studies in establishing associative, but not causative, relationships [26]. Similarly, with diabetic kidney disease, it is unlikely that kidney disease is a causative factor for PDR but rather reflects the same underlying physiologic processes related to microvascular damage. This association is consistent with several studies that have confirmed the association between diabetic nephropathy with proliferative diabetic retinopathy [27]. DR is known to be a significant and independent predictor of progression to micro- or macroalbuminuria, but it is unclear whether albuminuria increases the risk of DR [28]. A key area of further research is to determine whether diabetic nephropathy precedes retinopathy or vice versa. Regardless of the direction of causality, our findings emphasize the need for a low threshold for referrals for routine eye evaluations among patients with chronic kidney disease from diabetes. Current guidelines from the American Diabetes Association do not recommend more frequent monitoring for DR in patients with concurrent nephropathy, but increasing the frequency of retinopathy screening among these patients should be considered given the close association [29].

One of the strengths of using data from *All of Us* is that the program places an emphasis on enrolling minorities who are underrepresented in biomedical research [30]. Racial and ethnic minorities are not only more affected by complications of diabetic retinopathy, but also have lower rates of eye care utilization [31]. Our cohort was diverse; 15.2% of participants identified as Hispanic or Latino, 20.4% identified as Black, and 53.2% identified as female. In the future, with ongoing enrollment increasing cohort sizes in *All of Us*, more detailed investigation into healthcare disparities for specific sub-groups will be possible.

An additional strength of this study is that the *All of Us* database includes patient-reported responses to more than forty survey questions regarding healthcare access and utilization. This information is useful because the content of social history information in electronic health records is typically limited to drug and alcohol use, occupation, and living situation. Information about social determinants of health is typically limited or not recorded.

One limitation of the study is the inability to establish causal relationships due to the observational study design. In addition, cohort definitions relied on diagnostic billing codes and there is potential for misclassification or inconsistencies in diagnoses. For instance, we were not able to validate baseline DR severity, as the *All of Us* database does not currently provide images or clinical notes pertaining to patient eye exams. This limitation is common to analyses of healthcare claims data.

## Conclusion

Using a novel nationwide database, we found that DR patients have substantial barriers to healthcare access. In addition to financial and social determinants, race and religion discordance between providers and patients may increase the likelihood of PDR and related complications.

## Supporting information

S1 File (DOCX)

## Abbreviations and acronyms

*All of Us*: *All of Us* Research Program
CDR: Curated Data Repository
CI: Confidence Interval
CPT: Current Procedure Terminology
DR: Diabetic Retinopathy
EHRs: Electronic Health Records
ICD: International Classification of Diseases
LOINC: Logical Observation Identifiers Names and Codes
OHDSI: Observational Health and Data Sciences Initiative
OMOP: Observational Health and Medicines Outcomes Partnership
OR: Odds Ratio
PDR: Proliferative Diabetic Retinopathy
PM: Physical Measurements
SNOMED: Systematized Nomenclature of Medicine
SD: Standard Deviation

## References

[1] Kempen JH, O’Colmain BJ, Leske MC, Haffner SM, Klein R, Moss SE, et al. The Prevalence of Diabetic Retinopathy among Adults in the United States. Arch Ophthalmol. 2004;122(4). 10.1001/archopht.122.4.552 15078674

[2] Solomon SD, Chew E, Duh EJ, Sobrin L, Sun JK, VanderBeek BL, et al. Diabetic retinopathy: A position statement by the American Diabetes Association. Diabetes Care. 2017; 10.2337/dc16-2641 PMC5402875 28223445

[3] Paz SH, Varma R, Klein R, Wu J, Azen SP. Noncompliance with Vision Care Guidelines in Latinos with Type 2 Diabetes Mellitus. The Los Angeles Latino Eye Study. Ophthalmology. 2006;113(8). doi: 10.1016/j.ophtha.2006.04.018 16769120

[4] Villarroel MA, Vahratian A, Ward BW. Health care utilization among U.S. adults with diagnosed diabetes, 2013. NCHS Data Brief. 2015;(183). 25647399

[5] Zhang X, Andersen R, Saaddine JB, Beckles GLA, Duenas MR, Lee PP. Measuring access to eye care: A public health perspective. Vol. 15, Ophthalmic Epidemiology. 2008. doi: 10.1080/09286580802399102 19065435

[6] Willis JR, Doan Q V., Gleeson M, Haskova Z, Ramulu P, Morse L, et al. Self-reported healthcare utilization by adults with diabetic retinopathy in the United States. Ophthalmic Epidemiol. 2018;25(5–6). doi: 10.1080/09286586.2018.1489970 29958092

[7] The “All of Us” Research Program. N Engl J Med. 2019;

[8] Ramirez AH, Sulieman L, Schlueter DJ, Halvorson A, Qian J, Ratsimbazafy F, et al. The All of Us Research Program: Data quality, utility, and diversity. medRxiv. 2020; 10.1016/j.patter.2022.100570 PMC9403360 36033590

[9] All of Us Research Hub.

[10] Methods–All of Us Research Hub.

[11] Diabetic Retinopathy Data Exploration and Preprocessing Notebook [Internet]. 2020. Available from: https://workbench.researchallofus.org/workspaces/aou-rw-47ab97fd/duplicateofsocialdeterminantsandhealthcareaccessineyeconditions/notebooks/preview/Diabetic Retinopathy—Data Exploration and Preprocessing.ipynb

[12] Social Determinants and Healthcare Access All of Us Workspace [Internet]. Available from: https://workbench.researchallofus.org/workspaces/aou-rw-47ab97fd/duplicateofsocialdeterminantsandhealthcareaccessineyeconditions/data

[13] Social Determinants and Healthcare Access in Eye Conditions Workspace [Internet]. Available from: https://workbench.researchallofus.org/workspaces/aou-rw-0b6764fb/dupsocialdeterminantsandhealthcareaccessineyeconditionsv4dataset/data

[14] Saaddine JB, Honeycutt AA, Narayan KMV, Zhang X, Klein R, Boyle JP. Projection of diabetic retinopathy and other major eye diseases among people with diabetes mellitus: United States, 2005–2050. Arch Ophthalmol. 2008;126(12). doi: 10.1001/archopht.126.12.1740 19064858

[15] Hartnett ME, Key IJ, Loyacano NM, Horswell RL, DeSalvo KB. Perceived Barriers to Diabetic Eye Care: Qualitative Study of Patients and Physicians. Arch Ophthalmol [Internet]. 2005 Mar 1;123(3):387–91. Available from: doi: 10.1001/archopht.123.3.387 15767483

[16] Chou CF, Sherrod CE, Zhang X, Barker LE, Bullard KMK, Crews JE, et al. Barriers to eye care among people aged 40 years and older with diagnosed diabetes, 2006–2010. Vol. 37, Diabetes Care. 2014. 10.2337/dc13-1507 PMC4930070 24009300

[17] Patel MR, Resnicow K, Lang I, Kraus K, Heisler M. Solutions to Address Diabetes-Related Financial Burden and Cost-Related Nonadherence: Results From a Pilot Study. Heal Educ Behav. 2018;45(1). doi: 10.1177/1090198117704683 28443371 PMC5908467

[18] Kang H, Lobo JM, Kim S, Sohn MW. Cost-related medication non-adherence among U.S. adults with diabetes. Diabetes Res Clin Pract. 2018;143. doi: 10.1016/j.diabres.2018.06.016 29944967 PMC6204232

[19] Surendran TS, Raman R. Teleophthalmology in diabetic retinopathy. In: Journal of Diabetes Science and Technology. 2014. doi: 10.1177/1932296814522806 24876576 PMC4455409

[20] Avidor D, Loewenstein A, Waisbourd M, Nutman A. Cost-effectiveness of diabetic retinopathy screening programs using telemedicine: A systematic review. Vol. 18, Cost Effectiveness and Resource Allocation. 2020. doi: 10.1186/s12962-020-00211-1 32280309 PMC7137317

[21] Aguwa UT, Aguwa CJ, Repka M, Srikumaran U, Woreta F, Singman EL, et al. Teleophthalmology in the Era of COVID-19: Characteristics of Early Adopters at a Large Academic Institution. Telemed e-Health. 2021;27(7). 10.1089/tmj.2020.0372 33074795

[22] Ma A, Sanchez A, Ma M. The Impact of Patient-Provider Race/Ethnicity Concordance on Provider Visits: Updated Evidence from the Medical Expenditure Panel Survey. J Racial Ethn Heal Disparities. 2019;6(5). 10.1007/s40615-019-00602-y 31236800

[23] Takeshita J, Wang S, Loren AW, Mitra N, Shults J, Shin DB, et al. Association of Racial/Ethnic and Gender Concordance Between Patients and Physicians With Patient Experience Ratings. JAMA Netw Open [Internet]. 2020 Nov 9;3(11):e2024583–e2024583. Available from: doi: 10.1001/jamanetworkopen.2020.24583 33165609 PMC7653497

[24] Shen MJ, Peterson EB, Costas-Muñiz R, Hernandez MH, Jewell ST, Matsoukas K, et al. The Effects of Race and Racial Concordance on Patient-Physician Communication: A Systematic Review of the Literature. J Racial Ethn Heal Disparities. 2018;5(1). doi: 10.1007/s40615-017-0350-4 28275996 PMC5591056

[25] Fairless EA, Nwanyanwu KH, Forster SH, Teng CC. Ophthalmology Departments Remain Among the Least Diverse Clinical Departments at United States Medical Schools. In: Ophthalmology. 2021. 10.1016/j.ophtha.2021.01.006 PMC8328189 33440211

[26] Altman N, Krzywinski M. Association, correlation and causation. Nat Methods. 2015;12(10). doi: 10.1038/nmeth.3587 26688882

[27] Tzeng TF, Hsiao PJ, Hsieh MC, Shin SJ. Association of nephropathy and retinopathy, blood pressure, age in newly diagnosed type 2 diabetes mellitus. Kaohsiung J Med Sci. 2001;17(6). 11559967

[28] Chandy A, Pawar B, John M, Isaac R. Association between diabetic nephropathy and other diabetic microvascular and macrovascular complications. Saudi J Kidney Dis Transpl. 2008;19(6). 18974577

[29] Pearce I, Simó R, Lövestam-Adrian M, Wong DT, Evans M. Association between diabetic eye disease and other complications of diabetes: Implications for care. A systematic review. Vol. 21, Diabetes, Obesity and Metabolism. 2019. doi: 10.1111/dom.13550 30280465 PMC6667892

[30] Mapes BM, Foster CS, Kusnoor S V., Epelbaum MI, AuYoung M, Jenkins G, et al. Diversity and inclusion for the All of Us research program: A scoping review. PLoS ONE. 2020. doi: 10.1371/journal.pone.0234962 32609747 PMC7329113

[31] West SK, Klein R, Rodriguez J, Muñoz B, Broman AT, Sanchez R, et al. Diabetes and diabetic retinopathy in a Mexican-American population: Proyecto VER. Diabetes Care. 2001;24(7). 10.2337/diacare.24.7.1204 11423503

