# Correction: Associations between healthcare utilization and access and diabetic retinopathy complications using *All of Us* nationwide survey data

**DOI:** 10.1371/journal.pone.0285302

**Published:** 2023-04-27

**Authors:** Alison X. Chan, John J. McDermott IV, Terrence C. Lee, Gordon Y. Ye, Bita Shahrvini, Bharanidharan Radha Saseendrakumar, Sally L. Baxter

## Notice of Republication

This article was republished on April 18, 2023, to correct the abstract and Table 1 in the original publication that are in violation of the *All of Us* Data and Statistics Dissemination Policy. The authors apologize for their non-compliance with the policies and for any breach of trust this may have caused with *All of Us* participants. No *All of Us* participants were re-identified during the process of conducting the study; all data remained de-identified. The authors thank the *All of Us* program and its participants for making this research study possible and again apologize for this unintentional oversight.

Please download this article again to view the correct version.
