# Supplementary material for: Associations between healthcare utilization and access and diabetic retinopathy complications using All of Us nationwide survey data
Source: PLoS One. 2022 Jun 15;17(6):e0269231. doi: 10.1371/journal.pone.0269231 (PMC9200294; doi:10.1371/journal.pone.0269231)
Supplement: S1 File — (DOCX) [file pone.0269231.s001.docx]

**SUPPORTING INFORMATION**

**Supplemental Table 1.** Cohort definition of adults with type 2 diabetes mellitus diagnosed with diabetic retinopathy in *All of Us.* Participants with the following qualifying Systematized Nomenclature of Medicine Clinical Terms (SNOMED-CT) codes were included in the cohort.

| **SNOMED-CT Code** | **Description** |
| --- | --- |
| **4855003** | Retinopathy due to diabetes mellitus |
| **390834004** | Nonproliferative diabetic retinopathy |
| **422034002** | Retinopathy with type 2 diabetes mellitus |
| **312903003** | Mild nonproliferative diabetic retinopathy |
| **1551000119108** | Nonproliferative diabetic retinopathy due to type 2 diabetes mellitus |
| **1501000119109** | Proliferative diabetic retinopathy due to type 2 diabetes mellitus |
| **312904009** | Moderate nonproliferative diabetic retinopathy |
| **312905005** | Severe nonproliferative diabetic retinopathy |
| **399872003** | Severe nonproliferative diabetic retinopathy with clinically significant macular edema |
| **399873008** | Severe nonproliferative diabetic retinopathy with no macular edema |
| **769183005** | Mild nonproliferative diabetic retinopathy of right eye |
| **769184004** | Mild nonproliferative diabetic retinopathy of left eye |
| **408414006** | O/E - left eye proliferative diabetic retinopathy |
| **408413000** | O/E - right eye proliferative diabetic retinopathy |
| **312909004** | Proliferative diabetic retinopathy - iris neovascularization |
| **193349004** | Preproliferative diabetic retinopathy |
| **769188001** | Severe nonproliferative diabetic retinopathy of left eye |
| **769187006** | Severe nonproliferative diabetic retinopathy of right eye |
| **769186002** | Moderate nonproliferative diabetic retinopathy of left eye |
| **769185003** | Moderate nonproliferative diabetic retinopathy of right eye |

**Supplemental Table 2.** Factors evaluated in bivariate analyses of odds of developing proliferative diabetic retinopathy or related complications. Crude odds ratios (OR) and 95% confidence intervals are reported for each factor. Factors meeting statistical significance (p<0.05) have been highlighted in Table 3 of the main text.

|  | **Odds Ratio (OR)** | **2.5 %** | **97.5 %** | **p-value** |
| --- | --- | --- | --- | --- |
| Asian | 1.776 | 0.553 | 4.930 | 0.293 |
| Black or African American | 1.358 | 0.864 | 2.104 | 0.177 |
| Other | 0.947 | 0.553 | 1.569 | 0.837 |
| Gender | 0.941 | 0.654 | 1.353 | 0.745 |
| Hispanic or Latino | 0.901 | 0.527 | 1.482 | 0.692 |
| Age (years) | 0.983 | 0.968 | 0.999 | 0.034 |
| Delayed filling prescription to save money | 1.372 | 0.838 | 2.190 | 0.195 |
| Could not afford follow up care | 1.353 | 0.695 | 2.493 | 0.349 |
| Could not afford healthcare provider | 1.438 | 0.676 | 2.851 | 0.318 |
| Could not afford healthcare specialist | 1.599 | 0.864 | 2.840 | 0.120 |
| Skipped medication to save money | 2.156 | 1.291 | 3.532 | 0.003 |
| Took less medication to save money | 1.908 | 1.119 | 3.175 | 0.015 |
| Worried about paying | 1.251 | 0.976 | 1.599 | 0.075 |
| Delayed care due to inability to afford co-pay | 1.326 | 0.292 | 4.510 | 0.675 |
| Delayed care due to elderly care | 1.390 | 0.743 | 2.480 | 0.281 |
| Delayed care due to high deductible | 3.183 | 1.120 | 8.690 | 0.024 |
| Delayed care due to having to pay out of pocket | 1.389 | 0.728 | 2.519 | 0.295 |
| Delayed care because of feeling nervous | 1.680 | 1.010 | 2.728 | 0.040 |
| Delayed care due to living in rural area | 1.493 | 0.795 | 2.679 | 0.193 |
| Took less medication to save money | 2.075 | 1.009 | 4.075 | 0.039 |
| Delayed care due to inability to take time off work | 1.200 | 0.526 | 2.489 | 0.642 |
| Delayed care because of lack of transportation | 1.910 | 1.192 | 3.009 | 0.006 |
| Delayed care because health care provider differed from patient in race, religion, or native language | 1.167 | 0.795 | 1.650 | 0.401 |
| Importance of health care provider understanding or having similar race, religion, or native language as patient | 1.176 | 1.011 | 1.374 | 0.038 |
| Patient has spoken to a medical specialist in the past 12 months | 0.938 | 0.601 | 1.501 | 0.782 |
| Number of medical specialist visits in the past 12 months | 1.067 | 0.944 | 1.199 | 0.283 |
| Healthcare information was easy to understand | 1.049 | 0.785 | 1.422 | 0.749 |
| Patient Identified a Place for Health Advice | 1.378 | 0.885 | 2.123 | 0.151 |
| Patient has spoken to an eye doctor in the past 12 months | 1.580 | 0.895 | 2.985 | 0.134 |
| Number of eye doctor visits | 1.351 | 1.199 | 1.525 | 0.000 |
| Healthcare coverage compared to a year ago described as better, worse, or about the same | 1.170 | 0.853 | 1.651 | 0.349 |
| Told by a healthcare provider insurance was not accepted | 1.545 | 0.863 | 2.667 | 0.128 |
| Diagnosed with peripheral neuropathy due to diabetes mellitus | 0.990 | 0.050 | 6.752 | 0.993 |
| Diagnosed with kidney disorder due to diabetes mellitus | 2.025 | 1.323 | 3.067 | 0.001 |
| Creatinine (minimum value) | 1.397 | 0.987 | 2.060 | 0.066 |
| Creatinine (maximum value) | 0.999 | 0.989 | 1.005 | 0.803 |
| Creatinine (mean value) | 1.129 | 1.003 | 1.277 | 0.043 |
| A1c (minimum value) | 1.086 | 0.983 | 1.206 | 0.115 |
| A1c (maximum value) | 1.000 | 1.000 | 1.000 | 0.319 |
| A1c (mean value) | 1.000 | 1.000 | 1.000 | 0.273 |

**Supplemental Table 3.** Demographic characteristics of the *All of Us* diabetic retinopathy cohort, United States population (based on 2020 Census Data*), adult enrollment in the *All of Us* database, and the 2010 National Eye Institute diabetic retinopathy data and statistics^†^

|  | *All of Us* diabetic retinopathy cohort | United States Census | *All of Us* overall adult enrollment | 2010 Diabetic retinopathy statistics reported by the National Eye Institute |
| --- | --- | --- | --- | --- |
| Gender   Female  Male | 53.2% 46.8% | 51%  49% | 60% 40% | 51% 49% |
| Race  White  Black   Hispanic or Latino  Other | 60.6% 20.4% 15.2%  3.8% | 62.7% 12.1% 18.6%  6.6% | 68.1% 27.6% 2.5%  1.8% | 68% 11% 16% 5% |

* United States Census Bureau. https://www.census.gov/en.html. Published 2020.

† Diabetic Retinopathy Data and Statistics | National Eye Institute. Accessed December 29, 2021. <https://www.nei.nih.gov/learn-about-eye-health/outreach-campaigns-and-resources/eye-health-data-and-statistics/diabetic-retinopathy-data-and-statistics>

**Supplemental Table 4.** Missing values (count and percentage) for each variable

| **Variable** | **Number missing** | **Percentage missing (%)** |
| --- | --- | --- |
| Number of medical specialist visits | 289 | 39.643 |
| Number of eye doctor visits | 228 | 31.276 |
| Minimum A1c value | 198 | 27.16 |
| Maximum A1c value | 198 | 27.16 |
| Mean A1c value | 198 | 27.16 |
| Spoken to a medical specialist | 142 | 19.479 |
| Spoken to an eye doctor | 120 | 16.461 |
| Inability to afford specialist | 111 | 15.226 |
| Minimum creatinine value | 110 | 15.089 |
| Maximum creatinine value | 110 | 15.089 |
| Mean creatinine value | 110 | 15.089 |
| Inability to afford follow-up care | 108 | 14.815 |
| Delayed care due to providing care for others | 106 | 14.54 |
| Inability to afford healthcare provider | 102 | 13.992 |
| Delayed care due to high cost of deductible | 102 | 13.992 |
| Delayed care due to childcare | 100 | 13.717 |
| Delayed care due to inability to afford copay | 99 | 13.58 |
| Delayed care due to having to pay out of pocket | 97 | 13.306 |
| Delayed care due to inability to take time off work | 85 | 11.66 |
| Delayed care due to feeling nervous | 52 | 7.133 |
| Delayed care due to living in a rural area | 36 | 4.938 |
| Delayed or avoided care due to provider-physician discordance in race, language, or religion | 34 | 4.664 |
| Delayed filling a prescription to save money | 30 | 4.115 |
| Worried about paying | 24 | 3.292 |
| Healthcare coverage | 22 | 3.018 |
| Importance of healthcare provider concordance in race, language, or religion | 20 | 2.743 |
| Delayed care due to transportation | 19 | 2.606 |
| Took less medication to save money | 16 | 2.195 |
| Have a place to go to for health advice | 16 | 2.195 |
| Ease of understanding health materials | 15 | 2.058 |
| Insurance accepted by healthcare provider | 14 | 1.92 |
| Skipped medication to save money | 12 | 1.646 |

**Supplemental Table 5.** Diagnosis and procedure codes used to define the outcome of proliferative diabetic retinopathy and related complications. Systematized Nomenclature of Medicine Clinical Terms (SNOMED-CT) codes and Current Procedure Terminology (CPT) codes were used.

| **Code** | **Description** |
| --- | --- |
| SNOMED-CT 1501000119109 | Proliferative diabetic retinopathy due to type 2 diabetes mellitus |
| SNOMED-CT 59276001 | Proliferative retinopathy due to diabetes mellitus |
| SNOMED-CT 232086000 | Neovascular glaucoma |
| SNOMED-CT 232023006 | Tractional detachment of retina due to diabetes mellitus |
| SNOMED-CT 104951000119106 | Vitreous hemorrhage due to type 1 diabetes mellitus |
| SNOMED-CT 1491000119102 | Vitreous hemorrhage due to type 2 diabetes mellitus |
| SNOMED-CT 31341008 | Vitreous hemorrhage |
| CPT 67228 | Treatment of extensive or progressive diabetic retinopathy |
| CPT 67113 | Repair of complex retinal detachment (eg, proliferative vitreoretinopathy, stage C-1 or greater, diabetic traction retinal detachment, retinopathy of prematurity, retinal tear of greater than 90 degrees), with vitrectomy and membrane peeling, including |
| CPT 67040 | Vitrectomy, mechanical, pars plana approach; with endolaser panretinal photocoagulation |

**Supplemental Table 6.** Lab values, conditions associated with diabetic retinopathy, and demographic characteristics used as predictors in logistic regression modeling

| **Variable** | **Variable type** |
| --- | --- |
| **Lab values** |  |
| Creatinine value | Continuous |
| Glomerular filtration rate | Continuous |
| Fasting glucose level | Continuous |
| HbA1c measurement | Continuous |
| Microalbumin/creatinine in urine | Continuous |
|  |  |
| **Conditions associated with diabetic retinopathy** | |
| Peripheral neuropathy | Categorical |
| Macroalbuminuric nephropathy due to diabetes mellitus | Categorical |
| Microalbuminuric nephropathy due to diabetes mellitus | Categorical |
| Disorder of kidney due to diabetes mellitus | Categorical |
| Proteinuric nephropathy due to diabetes mellitus | Categorical |
|  |  |
| **Demographic characteristics** |  |
| Race/ethnicity | Categorical |
| Age | Continuous |
| Biological sex | Categorical |
| Gender | Categorical |
| Employment status (unemployed or employed) | Categorical |
| Annual household income | Continuous |
| Highest grade or year of school completed | Categorical |
| Own versus rent current home | Categorical |
| Health insurance coverage | Categorical |

**Appendix**
*All of Us* Research Program Survey on Healthcare Access and Utilization

**DURING THE PAST 12 MONTHS, were you told by a health care provider or doctor’s office that they did not accept your health care coverage?**

• Yes • No • Don’t know

**There are many reasons people delay getting medical care. Have you delayed getting care for any of the following reasons in the PAST 12 MONTHS?**

Didn’t have transportation.

• Yes • No • Don't know

You live in a rural area where distance to the health care provider is too far.

• Yes • No • Don't know

You were nervous about seeing a health care provider.

• Yes • No • Don't know

Couldn’t afford the copay.

• Yes • No • Don't know

Couldn’t get time off work.

• Yes • No • Don't know

You had to pay out of pocket for some or all of the procedure.

• Yes • No • Don't know

You provide care to an adult and could not leave him/her.

• Yes • No • Don't know

**DURING THE PAST 12 MONTHS, were any of the following true for you?**

You skipped medication doses to save money

• Yes • No • Don't know

You took less medicine to save money

• Yes • No • Don't know

You delayed filling a prescription to save money

• Yes • No • Don't know

**DURING THE PAST 12 MONTHS, was there any time when you needed any of the following, but didn't get it because you couldn't afford it?**

**To see a regular doctor or general health provider (in primary care, general practice, internal medicine, family medicine)**

• Yes • No • Don't know

To see a specialist

• Yes • No • Don't know

Follow-up care

• Yes • No • Don't know

**Is there a place that you USUALLY go to when you are sick or need advice about your health?**

• Yes

• There is NO place

• There is MORE THAN ONE place

• Don't know

**In regard to your health insurance or health care coverage, how does it compare to a year ago? Is it better, worse, or about the same?**

• Better

• Worse

• About the same

• Don't know

**The following questions are about your experiences with doctors and other health care providers in the past year. Some people think it is helpful if their providers are from the same background that they are – like in terms of race or religion or native language –because they think their doctors will better understand what they’re experiencing or going through.**

**How often have you either delayed or not gone to see doctors or health care providers because they were different from you in any of these ways?**

• Always

• Most of the time

• Some of the time

• None of the time

• Don't know

**DURING THE PAST 12 MONTHS, have you seen or talked to any of the following doctors or health care providers about your own health?**

**An optometrist, ophthalmologist, or eye doctor (someone who prescribes eyeglasses)?**

• Yes

• No

• Don’t know

If “yes” is selected, display the following:

**What is the total number of visits to an eye doctor that you made in the last 12 months?**

o 1

o 2-3

o 4-5

o 6-7

o 8-9

o 10-12

o 13-15

o 16 or more

o Don't know
